# Possibility of Enlargement in Left Medial Temporal Areas Against Cerebral Amyloid Deposition Observed During Preclinical Stage

**DOI:** 10.3389/fnagi.2022.847094

**Published:** 2022-04-19

**Authors:** Etsuko Imabayashi, Kenji Ishii, Jun Toyohara, Kei Wagatsuma, Muneyuki Sakata, Tetsuro Tago, Kenji Ishibashi, Narumi Kojima, Noriyuki Kohda, Aya M. Tokumaru, Hunkyung Kim

**Affiliations:** ^1^Research Team for Neuroimaging, Tokyo Metropolitan Institute of Gerontology, Tokyo, Japan; ^2^Diagnostic and Therapeutic Nuclear Medicine Group, Department of Molecular Imaging and Theranostics, Quantum Life and Medical Science Directorate, Institute for Quantum Medical Science, National Institutes for Quantum and Radiological Science and Technology, Chiba, Japan; ^3^School of Allied Health Sciences, Kitasato University, Sagamihara, Japan; ^4^Research Team for Promoting Independence and Mental Health, Tokyo Metropolitan Institute of Gerontology, Tokyo, Japan; ^5^Nutraceuticals Division, Otsu Nutraceuticals Research Institute, Otsuka Pharmaceutical Co., Ltd., Tokyo, Japan; ^6^Department of Radiology, Tokyo Metropolitan Institute of Gerontology, Tokyo, Japan

**Keywords:** Alzheimer’s disease, compensation, voxel-based morphometry, MRI, amyloid, preclinical, MCI

## Abstract

Neurodegenerative changes in the preclinical stage of Alzheimer’s disease (AD) have recently been the focus of attention because they may present a range of treatment opportunities. A total of 134 elderly volunteers who lived in a local community were investigated and grouped into preclinical and mild cognitive impairment stages according to the Clinical Dementia Rating test; we also estimated amyloid deposition in the brain using positron emission tomography (PET). A significant interaction between clinical stage and amyloid PET positivity on cerebral atrophy was observed in the bilateral parietal lobe, parahippocampal gyri, hippocampus, fusiform gyrus, and right superior and middle temporal gyri, as previously reported. Early AD-specific voxel of interest (VOI) analysis was also applied and averaged Z-scores in the right, left, bilateral, and right minus left medial temporal early AD specific area were computed. We defined these averaged Z-scores in the right, left, bilateral, and right minus left early AD specific VOI in medial temporal area as R-MedT-Atrophy-score, L-MedT-Atrophy-score, Bil-MedT-Atrophy-score, and R_L-MedT-Atrophy-score, respectively. It revealed that the R_L-MedT-Atrophy-scores were significantly larger in the amyloid-positive than in the amyloid-negative cognitively normal (CN) elderly group, that is, the right medial temporal areas were smaller than left in amyloid positive CN group and these left-right differences were significantly larger in amyloid positive than amyloid negative CN elderly group. The L-MedT-Atrophy-score was slightly larger (*p* = 0.073), that is, the left medial temporal area was smaller in the amyloid-negative CN group than in the amyloid-positive CN group. Conclusively, the left medial temporal area could be larger in CN participants with amyloid deposition than in those without amyloid deposition. The area under the receiver operating characteristic curve for differentiating amyloid positivity among CN participants using the R_L-MedT-Atrophy-scores was 0.73; the sensitivity and specificity were 0.828 and 0.606, respectively. Although not significant, a negative correlation was observed between the composite cerebral standardized uptake value ratio in amyloid PET images and L-MedT-Atrophy-score in CN group. The left medial temporal volume might become enlarged because of compensatory effects against AD pathology occurring at the beginning of the amyloid deposition.

## Introduction

Alzheimer’s disease (AD) is a progressive neurodegenerative disease that is histopathologically characterized by an accumulation of senile plaques consists of amyloid beta and neurofibrillary tangles consist of tau. According to the criteria published by the National Institute on Aging – Alzheimer’s Association (NIA-AA) in 2011, the concept of clinical stages of AD; preclinical AD, MCI due to AD, and AD dementia, are adopted as pathophysiological continuum with a temporal order of biomarker changes for amyloid beta and neurodegeneration. In 2018, NIA-AA updated and unified this guideline and labeled it as research framework (Jack et al., 2018). Within this research framework, tau was defined as an independent biomarker, and the diagnosis of AD was totally based on the biomarkers for amyloid beta, tau and neurodegeneration, but not on the stage of clinical symptoms. In this study, we studied the relationship between amyloid deposition measured with positron emission tomography (PET) and neurodegeneration as atrophy evaluated by magnetic resonance imaging (MRI). Amyloid deposition starts years before objective clinical cognitive impairment, continuous morphological medial temporal atrophy is reportedly appeared on MRI mainly with comparison in mild cognitive impairment (MCI) stage to cognitively normal controls (Zhang et al., 2021).

In the late 1980s, morphometry for AD was first attempted with manual tracing on MRI (Seab et al., 1988), followed by voxel-based volumetric analysis at the beginning of this century (Ohnishi et al., 2001). An automated voxel-based morphometry procedure was then developed around the same time as the first effective symptomatic treatment; since then, clinically automated procedures for medial temporal atrophy evaluation have progressed (Matsuda et al., 2012). In the early clinical stage of AD, atrophy begins in the medial temporal area, trans-entorhinal region, entorhinal cortex, and hippocampus (Arnsten et al., 2021). Atrophy in these areas can be automatically detected using Z-score analysis, which compares each individual image with healthy images from a database (Matsuda et al., 2012). We used the Voxel-based Specific Regional Analysis System for Alzheimer’s Disease (VSRAD^®^), which is Japanese-approved software using Statistical Parametric Mapping (SPM) 8 and the Diffeomorphic Anatomical Registration using Exponentiated Lie Algebra (DARTEL) algorithm for tissue segmentation and spatial normalization. VSRAD produces Z-score map after comparison to normal database preinstalled in the software and then calculates the mean Z-score within the bilateral, left, right, and right-minus-left early AD–specific voxel of interest (VOI). These disease-specific VOIs are the areas with statistically significant volume change between the early clinical stage of AD and normal control demarcated on the t-map obtained from group comparison, that is, data-driven optimized VOI. Analyses using these statistical masks are better than anatomical VOI at differentiating disease condition from disease-free condition because they reflect pathological condition (Herholz et al., 2002; Hirata et al., 2005).

Recently, neuromorphological changes associated with cognitive changes in the preclinical stages of AD have been investigated with amyloid positron emission tomography (PET) data. Amyloid deposition has been shown to occur more than 10 years before symptom onset, and these preclinical stages may provide a more effective opportunity for therapy (Sperling et al., 2011).

In this study, to evaluate volume change within the preclinical stage of AD influenced by amyloid deposition, voxel-based comparison of tissue-segmented MR images using volumetric methods was performed among four groups [amyloid-negative cognitively normal (CN), amyloid-positive CN, amyloid-negative with MCI, and amyloid-positive with MCI] to confirm the regional distribution of atrophy. Then, early AD-specific VOI analyses were performed on MRI data to mainly evaluate whether any early volume change could be detected even among CN participants at the preclinical stage within the area known to shrink during the early stage of symptomatic AD.

## Materials and Methods

### Participants

This was a prospective single-cohort study of volunteers who lived in Itabashi-ku, Tokyo. A total of 136 participants were recruited, all of whom lived independently. The Clinical Dementia Rating (CDR) and Mini Mental State Examination (MMSE) scales were administered, education duration was determined, and brain scans were performed for all participants. MRI scans were performed for screening and volumetry, and amyloid PET using ^18^F-flutemetamol was used to determine amyloid accumulation in the brain and to quantify the degree of amyloid accumulation.

### Image Scanning

Screening and volumetric MRIs were performed using Achieva 1.5T (Philips Healthcare, Andover, MA, United States). T1-weighted 3D turbo field echo SENSE1 sequence was used for volumetric MRI.

^18^F-Flutemetamol PET images were acquired using Discovery MI (GE Healthcare, Milwaukee, WI, United States) as a 30-min scan starting 90 min after injection of approximately 185 MBq of ^18^F-flutemetamol. Images were reconstructed using a 3D-ordered subset expectation maximization method with a time-of-flight procedure.

### Visual Interpretation of Amyloid Positron Emission Tomography Images

Two experts with more than 7 years of experience in visual interpretation of amyloid PET images interpreted the images according to the procedure approved by the US Food and Drug Administration^1^ : The brightness of the pons was adjusted to 90% of the maximum intensity of the color scale, and the accumulation of flutemetamol in five regions (frontal lobes, posterior cingulate and precuneus, lateral temporal lobes, inferolateral parietal lobes, and striatum) was evaluated. A scan was judged as positive if at least one region has gray matter radioactivity that is as intense as or exceeds the intensity of the adjacent white matter. Both experts were blinded to the participants’ information. When judgments were conflicting, both positive and negative, the final judgment was reached by consensus.

### Quantification of Amyloid Positron Emission Tomography Images

The CortexID Suite^2^ is a fully automated quantification method reported to agree well with the histopathologic classification of neuritic plaque density and has a strong concordance with visual read results (Thurfjell et al., 2014). The reference region was the pons, and the average standardized uptake value ratio (SUVR) within the composite VOIs was fully automatically computed from SUVR images that were spatially normalized to the Montreal Neurological Institute (MNI) template space (Thurfjell et al., 2014) as CortexID composite SUVR images (SUVR_CortexID_).

### Voxel-Based Morphometry

For voxel-based morphometry, the Computational Anatomy Toolbox 12 (CAT12)^3^ was used for image processing. The tissue segmentation procedure was initialized using standard tissue probability maps in SPM12^4^ and the DARTEL algorithm (Ashburner, 2007) was used.

### Statistical Analyses

#### Voxel-Based Analysis

A full factorial design was applied for voxel-based analysis using SPM12. A two-way factorial design with two factors was used: clinical stage (CN or MCI) and visual interpretation of amyloid PET accumulation (positive or negative). The interaction between clinical stage and amyloid PET positivity with the regional cortex volume variable was measured. Age and sex were removed from the comparison as nuisance variables, and total intracranial volume (TIV) was measured by summation of the brain cortex, white matter, and cerebrospinal fluid (CSF) space and used to correct inter-subject size variation through voxel-based analyses (Malone et al., 2015).

#### Voxel of Interest-Based *Z*-Score Analysis

*Z*-score analysis using a voxel-based specific regional analysis system for AD (VSRAD; Japanese Medical Device approval number 30200BZX00060000) was applied to 3D volumetric MR images. The operation achieved by VSRAD automatically is as follows: first, volumetry was performed using SPM8 and DARTEL, including tissue segmentation, spatial normalization, and smoothing. Second, the Z-score was calculated within each voxel compared with the normal database preinstalled within this software (Matsuda et al., 2012). Z-scores were calculated for each voxel using the following formula:


$$\mathrm{Z score}=\frac{\mathrm{mean}\mathrm{voxel}\mathrm{value}\mathrm{of}\mathrm{normal}\mathrm{database}-\mathrm{subject's voxel value}}{\mathrm{standard}\mathrm{deviation}\mathrm{of}\mathrm{normal}\mathrm{database}},$$

Where large Z-scores mean both smaller volume and severe atrophy.

**FIGURE 1:**
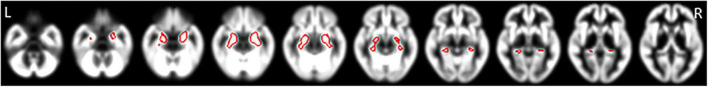
Specific VOIs for early AD in the medial temporal area of the brain cortex in the MNI space preinstalled in the VSRAD program. VOIs for early AD are drawn in red superimposed on segmented cortical T1-weighted MR images in the MNI space. Averaged positive *Z*-score values within each right, left, and bilateral VOI and the right-minus-left values were computed (Matsuda et al., 2012). We defined these Z-scores as R-MedT-Atrophy-score, L-MedT-Atrophy-score, Bil-MedT-Atrophy-score, and R_L-MedT-Atrophy-score, respectively. VOI, voxel of interest; AD, Alzheimer’s disease; MNI, Montreal Neurological Institute; VSRAD, Voxel-based Specific Regional Analysis System for Alzheimer’s Disease; MRI, magnetic resonance imaging.

Third, using specific VOIs for early AD in the medial temporal area in the MNI space, the VSRAD program automatically computed the averaged positive *Z*-score values within the right, left, bilateral and right-minus-left VOI values (Matsuda et al., 2012). In Figure 1, the VOIs for early AD in VSRAD are shown in red superimposed on the cortex in the MNI space. We defined averaged Z-score in this specific VOIs for early AD in the medial temporal area as MedT-Atrophy-score. The interaction between clinical stage and amyloid positivity on the averaged *Z*-score value in the right-specific VOI; R-MedT-Atrophy-scores, left-specific VOI; L-MedT-Atrophy-scores, bilateral-specific VOI; Bil-MedT-Atrophy-scores, and right-minus-left VOI; R_L-MedT-Atrophy-scores was statistically examined using EZR software (Saitama Medical Center, Jichi Medical University, Tochigi, Japan)^5^, which is a graphical user interface for R version 4.0.3 (The R Foundation for Statistical Computing, Vienna, Austria). More precisely, it is a modified version of R commander version 2.7–1 designed to add statistical functions frequently used in biostatistics (Kanda, 2013). All *p*-values were two-sided, with *p*-value ≤ 0.05 considered statistically significant. One-way analysis of variance and multiple comparisons were applied based on whether participants in each group (CN or MCI) were visually amyloid-positive or amyloid-negative and the *post hoc* test was applied with R software with age, sex and TIV as nuisance covariates. For the four groups (amyloid-positive CN, amyloid-positive MCI, amyloid-negative CN, and amyloid-negative MCI) in which significant differences in MedT-Atrophy-scores were observed even at the preclinical stage, the receiver operating characteristic (ROC) curve was plotted to estimate the clinical ability to differentiate amyloid positivity.

The Pearson product-moment correlation coefficients of SUVR_CortexID_ and MedT-Atrophy-scores and of education duration and MedT-Atrophy-scores were also computed using EZR. *p*-values ≤ 0.05 were considered statistically significant.

## Results

The MRI scans for screening and volumetry as well as amyloid PET scans were examined in 136 participants (female/male ratio, 96/40; mean age, 79.2 ± 4.0 years). Two participants, one whose volumetric MRI scan could not be segmented and another who had sequelae from a contusion in the temporal lobes, were excluded. The demographics of the remaining 134 participants are presented in Table 1. The global CDR score was used to determine the CN preclinical (CDR 0) or MCI stage (CDR 0.5 or 1.0) of AD. Among the 99 participants in the preclinical stage, 29 had positive amyloid PET scans and 70 had negative scans in the visual read. Among the 35 participants in the MCI group, 18 had positive scans and 17 had negative scans in the visual interpretation of amyloid PET images.

**TABLE 1:** Participants’ demographic characteristics.

	Total (*n* = 134)	Visually amyloid-positive		Visually amyloid-negative	
		CN stage (CDR 0) (*n* = 29)	MCI (CDR 0.5–1) (*n* = 18)	CN stage (CDR 0) (*n* = 70)	MCI (CDR 0.5–1) (*n* = 17)
Age (years)	79.18 ± 4.03	79.4 ± 4.66 (68–86)	80.3 ± 2.80^◆^ (75–86)	79.2 ± 4.10 (68–86)	77.6 ± 3.50^◆^ (69–84)
Female/male ratio	95:39	26:3	14:4	50:20	8:9
MMSE	27.80 ± 2.16	27.9 ± 2.17* (24–30)	26.1 ± 2.33* (22–30)	28.4 ± 1.63^☨^ (24–30)	26.9 ± 2.80^☨^ (22–30)
		27.2 ± 2.39^☆^		28.1 ± 1.99^☆^	
SUVR_CortexID_	0.517 ± 0.130	0.652 ± 0.115**	0.678 ± 0.119^§^	0.435 ± 0.0357^Ж^**	0.456 ± 0.0449^Ж§^
Education (years)	12.9 ± 2.51	12.6 ± 2.06	11.4 ± 2.62	13.4 ± 2.46	13.2 ± 2.81
		12.1 ± 2.34***		13.4 ± 2.34***	
TIV (mL)		1381 ± 134.9^Φ^	1385 ± 122.2^¶^	1446 ± 133.9^Φ^	1510 ± 142.1^¶^

*Values are presented as mean ± standard deviation (range) or ratio.*

Comparison between ^◆^amyloid-positive vs. amyloid-negative MCI, p = 0.0192. *Amyloid-positive CN vs. MCI, p = 0.00865. ☨ Amyloid-negative CN vs. MCI, p = 0.00449. ^☆^Amyloid-positive CN and MCI vs. amyloid-negative CN and MCI, p = 0.0300. **Amyloid-positive vs. amyloid-negative CN, p < 0.0001. ^§^Amyloid-positive and amyloid-negative MCI, p < 0.0001. ^¶^Amyloid-positive and amyloid-negative MCI, p = 0.00865. ^Ж^Amyloid-negative CN vs. MCI, p = 0.0378. ***Amyloid-positive vs. amyloid-negative CN and MCI, p = 0.00749. ^Φ^Amyloid-positive vs. amyloid-negative CN, p = 0.0309.

*CN, cognitively normal; MCI, mild cognitive impairment; CDR, Clinical Dementia Rating; MMSE, Mini Mental State Examination; SUVR, standardized uptake value ratio; TIV, total intracranial volume.*

Participants with MCI with visually interpreted amyloid PET positivity were significantly older than amyloid-negative participants with MCI. The MMSE scores were significantly lower in the MCI stage than in the preclinical stage for the amyloid-positive and amyloid-negative groups. However, the MMSE scores were not significantly different between patients in the amyloid-negative and amyloid-positive CN groups or between the amyloid-negative and amyloid-positive MCI groups. SUVR_CortexID_ was significantly higher in the visually amyloid-positive group. Within the amyloid-positive group, no significant difference in SUVR_CortexID_ was observed between the preclinical and MCI stages. Within the amyloid-negative group, those with MCI showed a significantly higher SUVR_CortexID_ than those at a preclinical stage. The visually amyloid-negative group had significantly more years of education than the amyloid-positive group. However, no significant differences were observed between the preclinical and MCI groups in this variable. The TIV, which consisted of the brain cortex, white matter, and CSF space, was significantly smaller in the visually amyloid-positive group than in the amyloid-negative group at both the preclinical and MCI stages. Accordingly, the TIV was measured and used to correct for different brain sizes and volumes.

**FIGURE 2:**
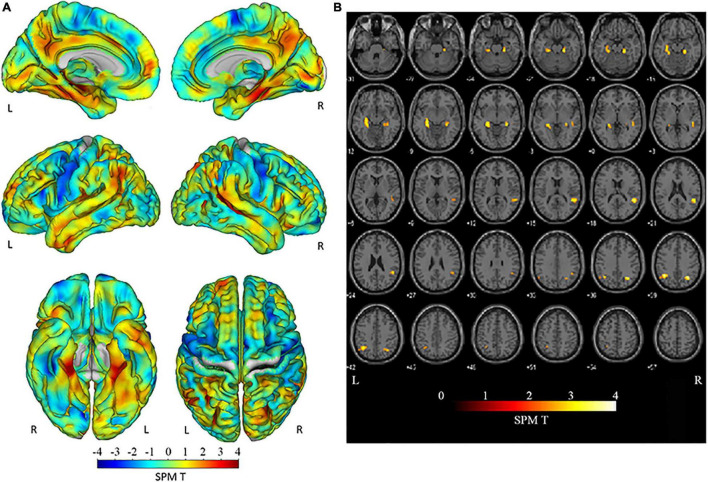
Negative interaction between clinical stages and amyloid positivity on brain cortex atrophy. **(A)** The SPM T map superimposed on the surface MR image in the MNI space showing atrophied areas both with clinical stage progression and amyloid positivity. Atrophy was observed in the parietal lobe, from the posterior cingulate to the precunei, from the middle to the posterior medial temporal area, and the left temporal tip. **(B)** Regions with statistically significant (*p* < 0.01; extent threshold, 300 voxels) atrophy in **(A)** superimposed on the template brain T1-weighted MR image in the MNI space. The anatomical labels of these statistically significantly atrophied areas, including the bilateral parietal lobe, parahippocampal gyri, hippocampus, fusiform, and right superior and middle temporal gyri, are presented in Table 2. Atrophy from the precuneus to the PCC and left temporal gyri was also observed but without significant differences. SPM, Statistical Parametric Mapping; MNI, Montreal Neurological Institute; PCC, posterior cingulate cortex.

Figure 2A shows the interaction between clinical stages and amyloid positivity in atrophy of the brain cortex. Figure 2B shows statistically significant (*p* < 0.01, extent threshold 300 voxels without multiple comparison) atrophied regions within Figure 2A superimposed on the template brain T1-weighted MR image in the MNI space. The anatomical labels of these statistically significantly atrophied areas are presented in Table 2 and include the bilateral parietal lobe, parahippocampal gyri, hippocampus, fusiform, and right superior and middle temporal gyri. Non-significant atrophy from the precuneus to the posterior cingulate cortex (PCC) and left temporal gyri was observed. No significant positive interaction was observed in the cerebral cortex; a significant positive interaction was observed only in the cerebellum.

**TABLE 2:** Areas of significant negative interaction between clinical stages and amyloid positivity on brain cortex atrophy observed in Figure 2A.

*T*-value	Size	*x*, *y*, and *z* (mm)	Area
4.34	358	33, −60, 38	Right angular gyrus
			Right superior and middle occipital
4.02	552	−39, −56, 39	Left angular gyrus
			Left superior and inferior parietal lobule
3.85	907	35, −36, −8	Right parahippocampal gyrus
			Right hippocampus
			Right fusiform
3.77	1152	54, −45, 18	Right superior and middle temporal gyrus
			Right angular gyrus
3.57	1556	−27, −36, −9	Left hippocampus
			Left parahippocampal gyrus
			Left fusiform

**FIGURE 3:**
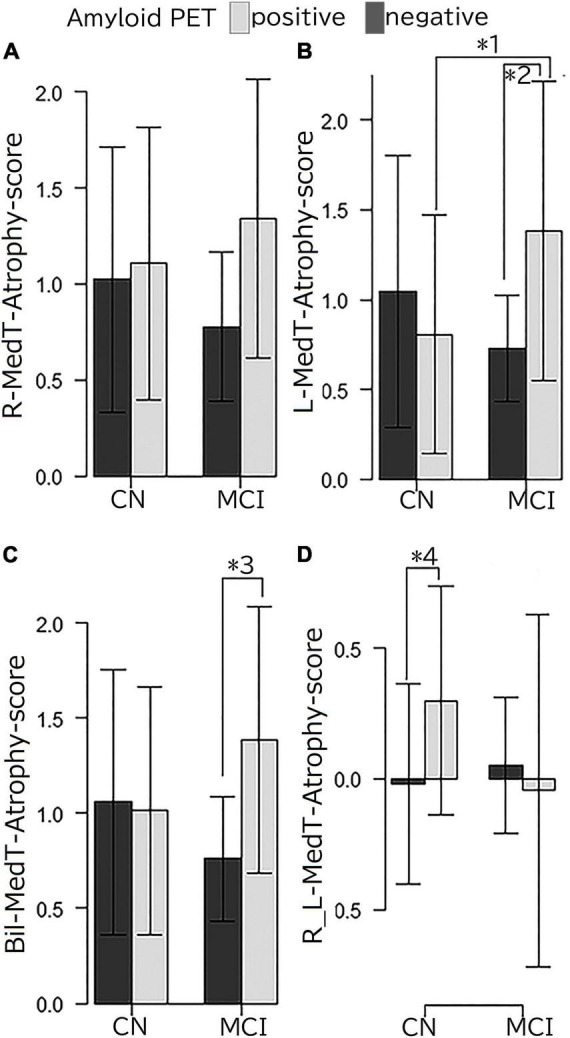
**(A)** R-MedT-Atrophy-scores, **(B)** L-MedT-Atrophy-scores, **(C)** Bil-MedT-Atrophy-scores, and **(D)** R_L-MedT-Atrophy-scores R, right; L, left; Bil, bilateral; R_L, right minus left; MedT-Atrophy-score, averaged *Z*-score values in early AD-specific medial temporal VOIs preinstalled in the Voxel-based Specific Regional Analysis System for Alzheimer’s Disease. Interaction of clinical stage and amyloid positivity in the mean *Z*-score value in the right-specific, left-specific, bilateral-specific, and R_L VOIs (*p* = 0.081, *p* = 0.0022, *p* = 0.013, and *p* = 0.019, respectively). Multiple comparison analysis after one-way analysis of variance using the Turkey-Kramer method with age, sex and TIV as nuisance variables had the following results: *1, *p* = 0.016; *2, *p* = 0.034; *3, *p* = 0.018; *4, *p* = 0.0013. Among the clinically normal participants, the L-MedT-Atrophy-scores were smaller in the amyloid-negative participants than in amyloid-positive ones, but the difference was not significant; the R_L-MedT-Atrophy-scores were significantly larger in the amyloid-positive participants than in the amyloid-negative participants.

The results of MedT-Atrophy-score analysis are shown in Figure 3. Interactions were observed among the L-MedT-Atrophy-scores (*p* = 0.0022), Bil-MedT-Atrophy-scores (*p* = 0.013), and R_L-MedT-Atrophy-scores (*p* = 0.019). Though any significant influence of age, sex, and TIV were not observed, we added them as nuisance variables and the *post hoc* test revealed significantly more severe atrophy in the amyloid-positive group than in the amyloid-negative group within the MCI stage on the L-MedT-Atrophy-scores (*p* = 0.034) and Bil-MedT-Atrophy-score (*p* = 0.018) as well as a significant difference between amyloid-positive and amyloid-negative groups within the preclinical stage on R_L-MedT-Atrophy-score (*p* = 0.0013). This significant laterality disappeared in the MCI group. Within the amyloid-positive group between CN and MCI, other significant differences were observed on the L-MedT-Atrophy-score (*p* = 0.016) and slight difference were observed on the R_L-MedT-Atrophy-score (*p* = 0.051).

**FIGURE 4:**
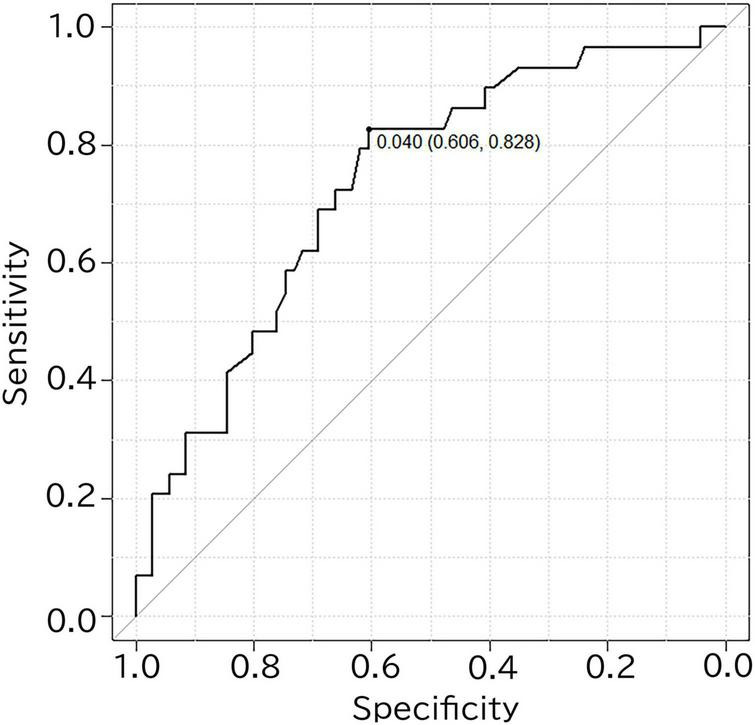
ROC curve for differentiating amyloid positivity in cognitively normal participants using the R_L-MedT-Atrophy-scores. The area under the curve, sensitivity, specificity and accuracy were 0.733 (95% confidence interval, 0.626–0.84) (DeLong et al., 1988), 0.828, 0.606, and 0.666, respectively. ROC, receiver operating curve; VOI, voxel of interest; R_L, right minus left; MedT-Atrophy-score, averaged *Z*-score values in early AD-specific medial temporal VOIs preinstalled in the Voxel-based Specific Regional Analysis System for Alzheimer’s Disease.

The performance for predicting visual amyloid positivity or negativity using R_L-MedT-Atrophy-score was quantified using an ROC curve. The area under the curve, sensitivity, specificity, and accuracy were 0.733 [95% confidence interval (CI), 0.626–0.84] (DeLong et al., 1988), 0.828, 0.606, and 0.666, respectively (Figure 4).

**FIGURE 5:**
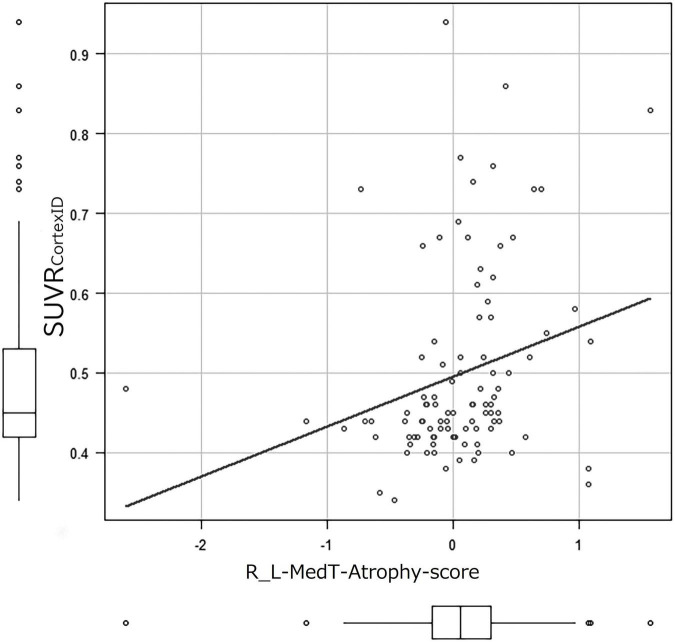
Correlation coefficient between SUVR_CortexID_ and MedT-Atrophy-score. A significant correlation was observed only in the R_L-MedT-Atrophy-scores at the preclinical stage (*p* = 0.00865). The Pearson’s product-moment correlation coefficient was 0.261 (95% confidence interval, 0.0683–0.435). R_L, right minus left; MedT-Atrophy-score, averaged *Z*-score values in early AD-specific medial temporal VOIs preinstalled in the Voxel-based Specific Regional Analysis System for Alzheimer’s Disease; SUVR_CortexID_, average standardized uptake value ratio within the composite VOIs in the Montreal Neurological Institute space obtained with the CortexID Suite.

The correlation coefficients of SUVR_CortexID_ and MedT-Atrophy-scores were only significant in the R_L-MedT-Atrophy-score in the preclinical stage (*p* = 0.00865). The Pearson product-moment correlation coefficient was 0.261 (95% CI, 0.0683–0.435) (Figure 5). Furthermore, a slight negative correlation was observed in the L-MedT-Atrophy-scores in the preclinical stage (*p* = 0.143). The Pearson product-moment correlation coefficient was −0.148 (95% CI, −0.334–0.0503) (Supplementary Figure 1).

**FIGURE 6:**
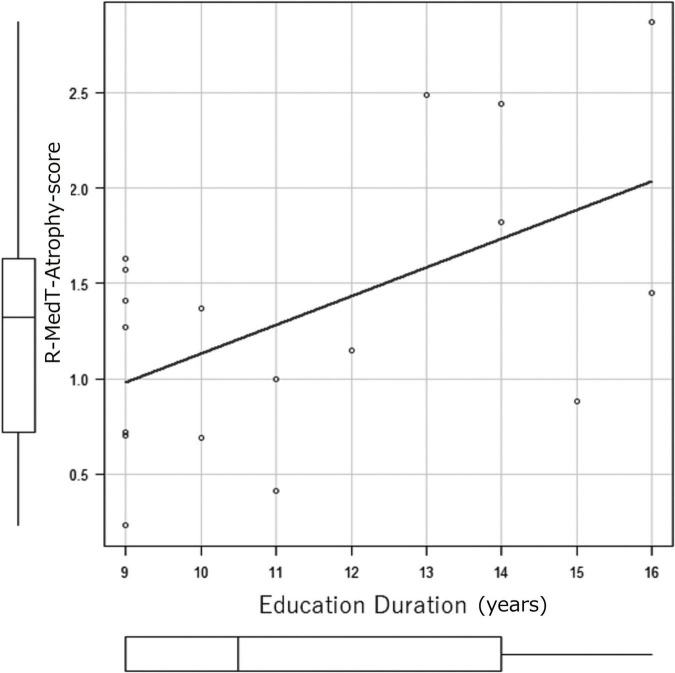
Correlation coefficient between education duration (years) and MedT-Atrophy-score. A significant correlation was observed only in the R-MedT-Atrophy-scores at the CN stage (*p* = 0.0196). The Pearson’s product-moment correlation coefficient was 0.544 (95% confidence interval, 0.103–0.806). R, right; MedT-Atrophy-score, averaged *Z*-score values in early AD-specific medial temporal VOIs preinstalled in the Voxel-based Specific Regional Analysis System for Alzheimer’s Disease.

The correlation coefficients of education duration and MedT-Atrophy-scores were only significant in the R-MedT-Atrophy-score in the MCI stage (*p* = 0.0196). The Pearson product-moment correlation coefficient was 0.544 (95% CI, 0.103–0.806) (Figure 6).

## Discussion

This cohort study evaluated volume change within the preclinical stage of AD influenced by amyloid deposition through ^18^F-flutemetamol amyloid PET and volumetric MRI on elderly participants living an ordinary life in a downtown area of Tokyo. In the voxel-based group analysis, significant atrophy in the medial temporal and lateral parietal was observed due to the interaction of stages and visually interpreted amyloid PET positivity. In the individual early AD-specific VOI analysis, a significant difference in R_L-MedT-Atrophy score was observed between the amyloid-positive and amyloid-negative participants, even in the CN stage.

In this study, the participants in the visually amyloid-positive MCI group were significantly older than those in the visually amyloid-negative MCI group. Younger elderly patients with MCI due to non-AD or older elderly patients with MCI due to AD might be easily included in this cohort study. On the contrary, older elderly patients with MCI due to non-AD or younger elderly patients with MCI due to AD might mainly refuse to participate in this study. Younger patients with MCI due to AD (i.e., early-onset AD) usually have more severe or progressive symptoms; thus, they might hesitate or refuse to attend a study recruiting normal participants. Alternatively, perhaps due to their heightened awareness of the disease, they might choose to go to the hospital rather than attend a study. Age and sex were removed from comparison as nuisance variables. The TIV was significantly smaller in the amyloid-positive group than in the amyloid-negative group at both preclinical and MCI stages. The TIV was used to correct for different brain sizes and volumes.

Brain atrophy was investigated cross-sectionally in the amyloid-negative and amyloid-positive groups during preclinical and MCI stages. Atrophy in the hippocampus and parahippocampal gyrus is a well-established finding in patients with MCI due to AD (Matsuda et al., 2012) and even in patients with early MCI (Grothe et al., 2016), which is consistent with our study. Only a loose criterion without multiple comparison could be used in our study, which we considered to be due to the inclusion of preclinical cases. Except for these regions, the lateral temporal areas showed atrophy in those with late MCI in the Alzheimer’s Disease Neuroimaging Initiative (ADNI) cohort (Grothe et al., 2016). In our study, except for the medial temporal area, atrophy in the lateral parietal regions was observed across the CN to the MCI stage according to the status of amyloid PET positivity or negativity (Figure 2B and Table 2). This difference might be attributed to the difference in the patients’ age between studies; participants in our study were older than those in the ADNI cohort (Grothe et al., 2016). Furthermore, cognitive reserve and education duration may have also resulted in bias. Bauckneht et al. (2018) reported hypometabolism in the parietal area in the highly educated group. The education duration in our study was shorter than that in the ADNI cohort but longer than that in the study of Bauckneht et al. (2018); furthermore, participants in our study showed more atrophy in the parietal area than those in the study of Bauckneht et al. (2018). Thus, depending on education and age, cognitive reserve might affect the distribution of atrophy.

Conclusively, medial temporal atrophy was observed in those with amyloid-positive MCI in this study. In a previous report, the medial temporal areas showed atrophy in patient with MCI due to AD with only clinical diagnosis and without amyloid confirmation (Matsuda et al., 2012). However, we confirmed this shrinkage in a more posterior lesion of the medial temporal area than those shown in a previous report (Matsuda et al., 2012) of amyloid deposition at an earlier stage.

MedT-Atrophy-scores were also computed in our study; data of participants in this study were compared with normal data set preinstalled in VSRAD using early AD-specific VOIs, which were also preinstalled in VSRAD. In the amyloid-positive group, the L-MedT-Atrophy-scores was significantly larger; that is, the medial temporal areas were significantly smaller or more atrophied in the MCI group than in the preclinical CN group. Concerning the size differences between the right and left medial temporal volumes in the preclinical stage, the R_L-MedT-Atrophy-scores were significantly larger in amyloid-positive than in amyloid-negative participants. This significant difference of R_L-MedT-Atrophy-scores between amyloid-positive and amyloid-negative groups in the preclinical CN stage was not observed in the MCI stage; moreover, the R_L-MedT-Atrophy-scores were slightly smaller in the amyloid-positive group at the MCI stage than in the group at the CN stage (*p* = 0.051). Furthermore, an additional ROC curve showed significant discrimination between amyloid-positive and amyloid-negative participants even in the preclinical stage using these R_L-MedT-Atrophy-scores (area under the curve, 0.733) (Figure 4). Larger R_L-MedT-Atrophy-scores indicated small L-MedT-Atrophy-scores or large R-MedT-Atrophy-scores. Thus, there can be two presumptions: either the left medial temporal volumes were larger in the amyloid-positive CN participants than in amyloid-negative CN ones or the right medial temporal volumes were smaller in the amyloid-positive CN participants than in amyloid-negative CN ones. According to Figures 3A,B, although the L-MedT-Atrophy-scores tended to be smaller in the amyloid-positive CN participants than in the amyloid-negative CN ones, and because significant results were observed only in the R_L-MedT-Atrophy-scores, both these factors could be related.

We assumed that a compensatory mechanism is involved in increasing the temporal volume. Medial temporal structures reportedly show increased perfusion (Alsop et al., 2008) or lesser atrophy than that observed in very mild to moderate AD (Matsuda et al., 2002; Guedj et al., 2009). Choi et al. (2021) reported that increased hippocampal glucose uptake was associated with the metabolic reconfiguration of microglia; they measured the triggering receptor expressed on myeloid cells 2 in the CSF. Furthermore, Fox et al. (2005) reported that Aβ immunization responders showed the whole brain and hippocampal volume reduction after 12 months of follow-up; however, the results were not significant and did not reflect worsening cognitive performance. According to these observations, not only medial temporal metabolism and perfusion but also volume change could be induced by immunization in the medial temporal area, and the volume could vary depending on function and inflammation. Regarding the core neuronal part of adult hippocampal neurogenesis (AHN), adult neurogenesis persists throughout adulthood in humans and decreases with age (Babcock et al., 2021), except for a niche part of AHN that includes characteristic extracellular matrix components, distinct vasculature structures, a host of secreted factors, and factors derived by microglia and astrocytes (Araki et al., 2021). AHN also decreases in patients with AD (Li Puma et al., 2020). Neurogenesis that contributes to the addition of new granule to the dentate gyri throughout the lifespan (although the efficiency decreases with age and in AD patients) has been observed in patients with AD as old as 90 years (Tobin et al., 2019). Furthermore, overexpression of human tau in mice reportedly induced AHN deficits but increased astrogenesis, which is associated with a downregulation of gamma-amino butyric acid in mice (Zheng et al., 2020). Hence, an observed increase in the left medial temporal volume could result from an increase in the niche of vascular and glial factors of AHN. This increase in the vascular factor may be attributed to glial cell activity, which may be essential for compensation and form the cognitive reserve.

Concerning the decrease in the right medial temporal volume, a pathology other than amyloid β, especially argyrophilic grain disease, could be considered. Adachi et al. (2010) reported that, in patients with argyrophilic grain stage 3 and CDR as 0, the right medial temporal volume was smaller; additionally, more pathologically extensive argyrophilic grains were observed predominantly in the right side. The mean age of their participants was 81 years. Contrarily, those with argyrophilic grain stage 3 and CDR > 0.5 had a dominant left side. Accordingly, those whose CDR was 0 and amyloid-positive participants in our cohort may have had such right-side dominant degenerative disease in the preclinical stage.

When the atrophy within the early AD-specific VOI of each case was individually compared with a normal database preinstalled in VSRAD using R_L-MedT-Atrophy-scores, we observed a significant difference between the amyloid-positive and amyloid-negative participants, even within the CN groups. Furthermore, when applying this R_L-MedT-Atrophy-scores as thresholds in the preclinical stage, the area under the curve for retrospective discrimination of amyloid positivity and negativity was 0.733. Supposedly, this value was sufficiently high to discriminate amyloid positivity and negativity using only volumetric MRI within the CN stage. We also found a significant correlation between R_L-MedT-Atrophy-scores and composite SUVR calculated with CortexID (SUVR_CortexID_). Accordingly, left medial temporal lesions were suspected to be enlarged due to the compensatory mechanism of the AHN.

Regarding the correlation between education duration and MedT-Atrophy-scores we hypothesized that if the right medial temporal area is more vulnerable and more atrophied for amyloid deposition than the left medial temporal area, the cognitive reserve in participants with long duration of education might maintain them in the MCI stage clinically, despite progression of shrinkage.

There is evidence of laterality in volume preservation in the medial temporal area that might correlate with compensation and cognitive reserve; however, this study has some limitations. The results were from only one cohort; hence, another cohort is needed to ensure such volume preservation and discuss compensation. Furthermore, in terms of brain and sex difference, our study included more women than men; thus, our result might be mostly representative of female sex. As the cohort in the present study was very small to compare female and male, comparison between male and female should be performed in a study with a larger cohort.

## Data Availability Statement

The original contributions presented in the study are included in the article/Supplementary Material, further inquiries can be directed to the corresponding author/s.

## Ethics Statement

The studies involving human participants were reviewed and approved by the Ethical Committee of the Tokyo Metropolitan Institute of Gerontology. The patients/participants provided their written informed consent to participate in this study.

## Author Contributions

HK designed and coordinated the study. NoK coordinated the study. EI performed the neuroimaging analysis, interpreted the neuroimaging data, and drafted the manuscript. KIi interpreted the imaging data and drafted the manuscript. AT clinically interpreted the neuroimaging data, designed the neuroimaging protocols, and reviewed the manuscript. JT and TT produced and qualified the tracer. KW performed and qualified the PET imaging data. MS performed the statistical analysis and reviewed the manuscript. KIb and NaK performed cognitive assessments and reviewed the manuscript. All authors contributed to the article and approved the submitted version.

## Conflict of Interest

NoK was a permanent employee of Otsuka Pharmaceutical Co., Ltd. The remaining authors declare that the research was conducted in the absence of any commercial or financial relationships that could be construed as a potential conflict of interest.

## Publisher’s Note

All claims expressed in this article are solely those of the authors and do not necessarily represent those of their affiliated organizations, or those of the publisher, the editors and the reviewers. Any product that may be evaluated in this article, or claim that may be made by its manufacturer, is not guaranteed or endorsed by the publisher.

## Footnotes

1
https://www.accessdata.fda.gov/drugsatfda_docs/label/2017/203137s008lbl.pdf

2
https://www.gehealthcare.co.uk/-/media/13c81ada33df479ebb5e45f450f13c1b.pdf

3
http://www.neuro.uni-jena.de/cat/

4
https://www.fil.ion.ucl.ac.uk/spm/software/spm12/

5
https://www.jichi.ac.jp/saitama-sct/SaitamaHP.files/statmedEN.html

## Supplementary Material

Supplementary Figure 1 Correlation coefficient between SUVR_CortexID_ and MedT-Atrophy-score. A non-significant negative correlation was observed in the L-MedT-Atrophy-score in the preclinical stage (*p* = 0.143). The Pearson’s product-moment correlation coefficient was −0.148 (95% confidence interval, −0.334–0.0503). L, left; MedT-Atrophy-score, averaged *Z*-score values in early AD-specific medial temporal VOIs preinstalled in the Voxel-based Specific Regional Analysis System for Alzheimer’s Disease; SUVR_CortexID_, average standardized uptake value ratio within the composite VOIs in the Montreal Neurological Institute space obtained with the CortexID Suite.

Supplementary Figure 2 The images to show representations of visually amyloid-negative **(A)** and visually amyloid-positive **(B)**. **(A)** This negative scan shows lower intensity in cortical gray matter than in white matter, creating clear gray-white matter contrast. **(B)** This positive scan have five cortical regions (frontal lobes, lateral temporal lobes, inferolateral parietal lobes, posterior cingulate and precuneus and striatum) in which gray matter signal is as intense or exceeds the intensity in adjacent white matter.
