# Supplementary figures and images for: Possibility of Enlargement in Left Medial Temporal Areas Against Cerebral Amyloid Deposition Observed During Preclinical Stage
Source: Front Aging Neurosci. 2022 Apr 19;14:847094. doi: 10.3389/fnagi.2022.847094 (PMC9063485; doi:10.3389/fnagi.2022.847094)

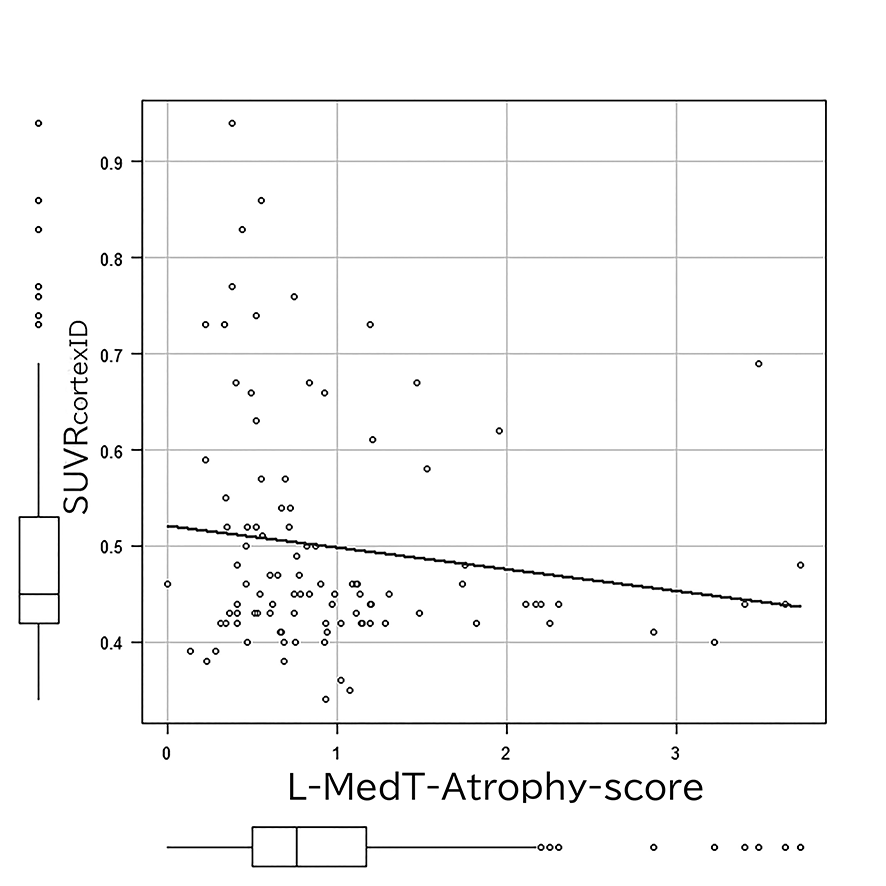

Supplement: Supplementary Figure 1 — Correlation coefficient between SUVRCortexID and MedT-Atrophy-score. A non-significant negative correlation was observed in the L-MedT-Atrophy-score in the preclinical stage (p = 0.143). The Pearson’s product-moment correlation coefficient was −0.148 (95% confidence interval, −0.334–0.0503). L, left; MedT-Atrophy-score, averaged Z-score values in early AD-specific medial temporal VOIs preinstalled in the Voxel-based Specific Regional Analysis System for Alzheimer’s Disease; SUVRCortexID, average standardized uptake value ratio within the composite VOIs in the Montreal Neurological Institute space obtained with the CortexID Suite. [file Image_1.TIF]

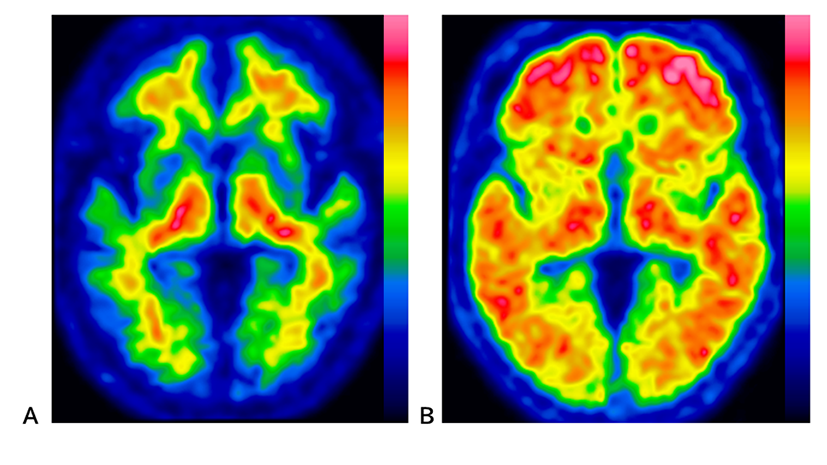

Supplement: Supplementary Figure 2 — The images to show representations of visually amyloid-negative (A) and visually amyloid-positive (B). (A) This negative scan shows lower intensity in cortical gray matter than in white matter, creating clear gray-white matter contrast. (B) This positive scan have five cortical regions (frontal lobes, lateral temporal lobes, inferolateral parietal lobes, posterior cingulate and precuneus and striatum) in which gray matter signal is as intense or exceeds the intensity in adjacent white matter. [file Image_2.TIF]
